# Efficacy of a progressive walking program and glucosamine sulphate supplementation on osteoarthritic symptoms of the hip and knee: a feasibility trial

**DOI:** 10.1186/ar2932

**Published:** 2010-02-12

**Authors:** Norman TM Ng, Kristiann C Heesch, Wendy J Brown

**Affiliations:** 1 The University of Queensland, School of Human Movement Studies, Blair Drive, St Lucia Campus, Brisbane, Queensland 4072, Australia; 2 Queensland University of Technology, School of Public Health, Victoria Park Road, Kelvin Grove, Brisbane, Queensland 4059, Australia

## Abstract

**Introduction:**

Management of osteoarthritis (OA) includes the use of non-pharmacological and pharmacological therapies. Although walking is commonly recommended for reducing pain and increasing physical function in people with OA, glucosamine sulphate has also been used to alleviate pain and slow the progression of OA. This study evaluated the effects of a progressive walking program and glucosamine sulphate intake on OA symptoms and physical activity participation in people with mild to moderate hip or knee OA.

**Methods:**

Thirty-six low active participants (aged 42 to 73 years) were provided with 1500 mg glucosamine sulphate per day for 6 weeks, after which they began a 12-week progressive walking program, while continuing to take glucosamine. They were randomized to walk 3 or 5 days per week and given a pedometer to monitor step counts. For both groups, step level of walking was gradually increased to 3000 steps/day during the first 6 weeks of walking, and to 6000 steps/day for the next 6 weeks. Primary outcomes included physical activity levels, physical function (self-paced step test), and the WOMAC Osteoarthritis Index for pain, stiffness and physical function. Assessments were conducted at baseline and at 6-, 12-, 18-, and 24-week follow-ups. The Mann Whitney Test was used to examine differences in outcome measures between groups at each assessment, and the Wilcoxon Signed Ranks Test was used to examine differences in outcome measures between assessments.

**Results:**

During the first 6 weeks of the study (glucosamine supplementation only), physical activity levels, physical function, and total WOMAC scores improved (*P *< 0.05). Between the start of the walking program (Week 6) and the final follow-up (Week 24), further improvements were seen in these outcomes (*P *< 0.05) although most improvements were seen between Weeks 6 and 12. No significant differences were found between walking groups.

**Conclusions:**

In people with hip or knee OA, walking a minimum of 3000 steps (~30 minutes), at least 3 days/week, in combination with glucosamine sulphate, may reduce OA symptoms. A more robust study with a larger sample is needed to support these preliminary findings.

**Trial Registration:**

Australian Clinical Trials Registry ACTRN012607000159459.

## Introduction

Osteoarthritis (OA) is the most common musculoskeletal disorder and the leading cause of pain and disability in the USA and Australia [1,2]. In Australia, it affects 7.8% of the population, and projections indicate that the prevalence will increase to 9.8% by 2020 [3].

There is no known cure for OA. The goal of treatment, therefore, is to help reduce patients' pain, prevent reductions in their functional ability and maintain or increase their joint mobility. For individuals with moderate symptoms of OA and no other health problems, international guidelines for initial treatment recommend non-pharmacological treatments, including lifestyle changes [4-9]. A number of non-pharmacological treatments have been studied for the management of OA, but because there have been few well-conducted studies, the effectiveness of most non-pharmacological treatments is open to question [10].

Exercise, however, as a treatment for OA has been studied in numerous randomised controlled trials, mostly in people with OA of the knee. Most of these have focused on improving the stability of joints, range of movement and aerobic fitness in order to decrease patients' pain and disability [11]. Patients with mild to moderate symptoms of knee or hip OA who have participated in aerobic exercise programs have experienced increases in aerobic capacity [11,12] and functional ability [13,14], and decreases in pain, fatigue, depression and anxiety [11-13,15]. These results have led to recommendations for the use of aerobic exercise for the treatment of OA [4,7-9].

A recent review of randomised controlled trials in patients with knee OA found three types of exercise program (supervised individual, supervised group-based and unsupervised home-based) have been evaluated, with decreases in pain and physical function not differing significantly among participants in the three types [13]. In contrast to pharmacological treatments, which can cause gastrointestinal side effects [16], moderate-intensity aerobic exercises are well tolerated over the long term and have similar effects (effect size [ES] = 0.52) [17] for reducing pain to those seen with paracetamol and nonsteroidal anti-inflammatory drugs (NSAIDs; ES = 0.32) [18]. Compared with supervised programs, home-based programs are more convenient for participants, feasible in community settings and cost-effective for large populations, suggesting their suitability as a public health approach [13].

Walking may be an appropriate activity for home-based programs [19], because it has resulted in greater improvements in pain and greater participation rates than other forms of aerobic exercise, such as running or cycling [20]. In studies assessing the effectiveness of walking for patients with knee OA, moderate improvements in pain (ES = 0.52) and physical functioning (ES = 0.32) have been found [17] without adverse effects on OA symptoms [14]. The Physical Activity Guidelines Advisory Committee recommends that individuals with OA engage in moderate-intensity, low-impact activities such as walking, three to five times per week for 30 to 60 minutes per session [21].

Despite the accumulating international evidence suggesting that aerobic exercise is effective in reducing symptoms of OA of the knee, and to a lesser degree of the hip, an important question remains: What is the appropriate 'dose' of exercise (intensity, frequency, and duration) for significant improvements in symptoms of knee and hip OA? More broadly, the question of an appropriate dose of exercise has yet to be determined for people with arthritis in general [21]. In previous studies, exercise format, duration, intensity, and type of exercise varied widely, making it difficult to specify the required dose for optimal benefits. Even among the studies that used walking, programs have varied in content, duration of sessions and length of the intervention [17]. Only one small study [22] has examined the dose issue, and it focused on intensity of exercise. The researchers found that higher and lower intensity exercises are equally effective in improving symptoms of OA.

One treatment that is used in combination with or without exercise by some people with early hip or knee OA is glucosamine sulphate (GS), a natural occurring substance believed to assist with building and repair of cartilage. It is taken as a complementary medicine that is safe and has few side effects [8]. Two recent randomised trials from Europe have shown that GS slows radiological progression of knee OA [23,24]. In a meta-analysis of 20 double-blind randomised control trials, glucosamine was reported to improve well-being and to be as safe as placebo [25]. Although results of a review further suggest glucosamine offers moderate improvements in well-being [26], some trials reported little or non-significant effects of glucosamine when compared with placebo [27,28]. These conflicting results could be due to differences in the type of preparation used (GS or glucosamine hydrochloride), dose or bioavailability of the glucosamine preparation used.

Although some individuals with OA are using both glucosamine and exercise to relieve symptoms, no study has examined the effectiveness of the combined effects of exercise and GS on relieving symptoms of hip and knee OA. The main aim of this feasibility study was to evaluate the combined effects of a progressive walking program and GS intake on symptoms of OA and physical activity participation in people with hip or knee OA. Secondary aims were to compare the effectiveness of two frequencies of walking (three and five days per week) and three step levels (1500, 3000 and 6000 steps per day) of walking, combined with GS intake, and to examine compliance with GS intake and the walking program.

## Materials and methods

### Participants

Adults with hip or knee OA were recruited in Brisbane, Australia, from flyers posted at community sites and in doctors' offices, newspaper and newsletter advertisements, and segments on local television and radio programs. Eligibility criteria were: aged 40 to 75 years; having physician-diagnosed OA in at least one hip or knee (verified by a doctor's letter confirming diagnosis); experiencing pain, stiffness, crepitus and difficulty with daily activities within the previous month; an ability to walk at least 15 minutes continuously; and an ability to safely participate in moderate-intensity exercise, as determined by the Sports Medicine Australia Stage I pre-exercise screening questions [29]. Individuals were excluded if they: had other forms of arthritis; had corticosteroid or viscosupplement injections within the previous three months; had a history of infection in a knee or hip; were living in a dependent environment; were taking daily medication for OA, including analgesia; or were allergic to shellfish. Individuals who were planning to have surgery in the next six months, receiving psychiatric or psychological treatment, pregnant or planning to become pregnant, exercising more than 60 minutes per week, or participating in another research study were also excluded.

### Study design

**Figure 1:**
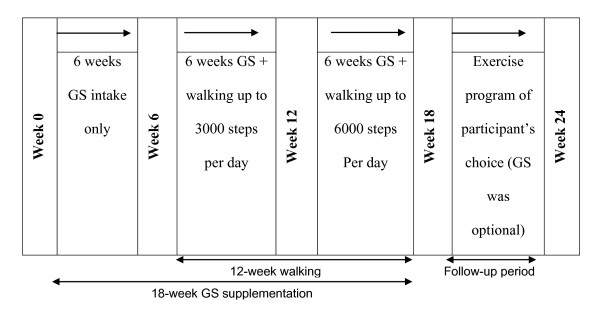
**Study design**. GS, glucosamine sulphate.

The study design is shown in Figure 1. This was a 24-week feasibility study with participants randomised to one of two intervention groups. Written informed consent was required at the baseline assessment, before participation could begin. Participants went through a two-week run-in, washout period before the first assessment. For this period and the rest of the study period, participants were informed to discontinue all over-the-counter or prescription medications for their OA symptoms. However, they were told that they could take their choice of rescue analgesia as needed for pain or swelling during the study period.

Before the first assessment, the data collector (author NTMN) used a computer random number generator to allocate participants to one of two groups. Participants were told of their group allocation at the baseline assessment. For practical reasons, allocation to group was not concealed. All participants received six-week supplies of GS at baseline, Week 6 and Week 12. At Week 6, participants began a 12-week progressive walking program called Stepping Out, either walking three or five days per week, depending on group assignment. The walking program ended at Week 18. The next six weeks constituted a follow-up period to test whether the intervention effects persisted after intervention completion. Study measures were administered during one-on-one interviews with participants at baseline and 6-, 12-, 18-, and 24-weeks after baseline. Assessments were conducted at the University of Queensland or at the participant's home. The study protocol was approved by the University of Queensland Medical Research Ethics Committee.

#### Main outcome measures

##### Physical activity

Time spent in physical activities was measured using a print version of the Active Australia physical activity questions [30], which have been shown to have acceptable reliability and validity [31]. A comparison of activity classification (i.e. 'active,' 'insufficiently active,' 'sedentary') showed moderate agreement between two testing occasions, 24 hours apart (Kappa coefficient = 0.50), a finding similar to those observed for other physical activity questionnaires used internationally [32]. Walking (to and from places and for exercise), leisure-time moderate-intensity physical activities, and vigorous-intensity physical activities were assessed separately. Minutes per week spent in each of these activities was summed to create a total physical activity score.

##### Osteoarthritis symptoms

The Western Ontario and McMaster Universities (WOMAC) Osteoarthritis Index numeric rating scale (NRS) 3.1 was used to measure pain, stiffness and physical function [33]. The index has been extensively validated and widely used in studies of knee and hip OA [34,35]. The index consists of three subscales with a total of 24 items (5 pain, 2 stiffness and 17 physical function) with test-retest reliability estimates of 0.68, 0.68 and 0.72 for the pain, stiffness, and physical function subscales, respectively [34,35]. Participants placed an 'x' on a numerical (visual analogue) scale ranging from 0 to 10. For the pain subscale, response options ranged from no pain to extreme pain; for the stiffness subscale, from no stiffness to extreme stiffness; and for the physical function subscale, from no difficulty to extreme difficulty. Responses to items on each of the three subscales were summed to create subscale scores. A total scale score (range 0 to 240) was calculated by simple summation of these subscale scores with higher scores indicating more severe symptoms.

Physical function was also assessed objectively with the Self-Paced Step Test (SPS) [36]. This test was selected because it could be used in participants' homes: it was portable, practical for use with minimal space and suitable for use in individuals with OA. Participants were asked to step up and down two 20 cm steps, 20 times at a comfortable pace. Time taken to complete the test was recorded to the nearest second with a digital stopwatch. A higher score indicated lower physical function. Immediately after the SPS test, the WOMAC pain subscale was re-administered to assess the level of pain after an activity that involved movement of the hip and knee joints.

#### Secondary outcome measures

##### Correlates of physical activity

Five theoretical constructs that were addressed in the Stepping Out program were measured with questionnaires. The Arthritis Self-Efficacy Scale assessed confidence of affecting change for managing arthritis pain, function and other symptoms, with higher scores indicating greater efficacy for managing symptoms [37]. One study has demonstrated adequate internal consistency for the scale's pain (Cronbach alpha = 0.76), function (Cronbach alpha = 0.89) and other symptoms (Cronbach alpha = 0.87) subscales [37]. The Self-Regulation Scale assessed the use of self-monitoring and goal setting strategies for physical activity behaviour with higher scores representing higher self-efficacy in meeting physical activity goals. Higher self-regulation scores have been associated with engaging in more moderate and vigorous physical activities (r = 0.50) [38]. The Self-Efficacy for Physical Activity Scale evaluated confidence in ability to participate regularly in physical activities, with higher scores indicating greater self-efficacy for physical activity. A high test-retest reliability estimate (r = 0.90) has been reported for this scale [39]. The Benefits of Physical Activity Scale determined whether participants were aware of the benefits of physical activity, and the Barriers to Physical Activity Scale identified factors that made participation in physical activities difficult [40]. Higher scores on the Benefits of Physical Activity Scale indicated a perception of more benefits, and a high test-retest reliability (*r *= 0.85) has been reported for this scale [40]. Higher scores on the Barriers to Physical Activity Scale indicated a perception of more barriers to physical activity. Barrier scale scores have been significantly and inversely correlated with exercise (r = -0.22) [40].

#### Health outcomes

The Goldberg Anxiety and Depression Scale [41] was used to measure symptoms of anxiety and depression. Nine items measured anxiety, and an additional nine measured depression, with response options of 'Yes' and 'No'. The summary score was calculated by adding the total number of 'Yes' responses to the 18 items. With a range of 0 to 18 on the scale, a higher score indicated more symptoms of anxiety and depression. The anxiety and depression subscales have sensitivities of 82% and 85%, respectively.

Body weight was measured to the nearest 0.5 kg using calibrated portable scales (SECA, Hamburg, Germany).

#### Demographic characteristics

Data on age, country of birth (a measure of race/ethnicity), marital status, living arrangements, caring responsibilities, education and employment status were collected using a self-report survey.

### The intervention

Starting at baseline, participants were supplied with GS (Bio-Organics™ Glucosamine Sulphate Complex 1000, Virginia, Queensland, Australia) and asked to take two capsules (750 mg each) daily. The Stepping Out program commenced at Week 6. It was developed to influence self-efficacy (confidence in one's ability to be physically active) and other constructs from Social Cognitive Theory that were hypothesised to impact self-efficacy [42]. This theory has been found to be effective as a framework for previous interventions in which OA sufferers managed their OA with exercise [43-48].

The Stepping Out program included: a walking guide; a pedometer; weekly log sheets for recording daily step counts, GS intake and intake of other medications and supplements; and a weekly planner for scheduling walking sessions (Table 1). Participants were encouraged to use strategies from the Stepping Out walking guide, to increase their self-efficacy towards walking. Strategies included behavioural contracting (using a written contract to meet the study requirements), goal setting, planning for walking sessions, and obtaining social support for walking. The interventionists also brainstormed with participants ways to increase their walking, make their walks enjoyable and overcome barriers to walking. This interaction with the interventionist lasted approximately one hour. Details of the content of each strategy can be found in Table 1. All participants received the same materials and instructions, but participants in the three-day walking group were asked to walk three days per week and participants in the five-day walking group were asked to walk five days per week.

**Table 1:** Stepping Out program topics and the theoretical constructs addressed by each one

Mode of delivery^a^	Topic	Content	Constructs addressed
Walking guide; one-on-one consultations	Provide opportunities and social support; correct misperceptions	Provide tips on finding opportunities in the environment for walking; Discuss barriers to doing the program and ways to overcome them in the future; Discuss walking as an activity readily available (e.g., can walk anyway, inexpensive); Suggest that friends or family be asked to provide encouragement and support for doing the program.	Environment
Walking guide; one-on-one consultations	Provide opportunities for experiencing benefits and learning what to expect from changing behaviour	Address health benefits of walking and other physical activities for OA sufferers; Explain normal bodily responses to starting a walking program; Provide warning signs of excessive exercise.	Outcome expectations
Walking guide	Rewarding for behaviour change	Discuss positive impact of walking on OA symptoms; Describe physiological benefits of walking as rewards for increasing walking behaviour.	Reinforcement
Walking guide; one-one-one consultations	Behavioural capability Mastery learning Observational learning	Discuss and demonstrate proper walking techniques pertaining to posture, arm motion, taking a step, walking stride, and pace; Discuss 'safe' walking; Advice on selecting walking shoes; Discuss the use of short bouts (1500 steps) of walking to improve health and OA symptoms; Instruct to increase steps at own rate; Display stretching exercises.	Self-efficacy
Walking guide; pedometer; log sheets; weekly planners; one-one consultations	Self-regulation and self-monitoring	Provide use of a pedometer for 12 weeks; Advice on and review of setting step goals; Guide in writing weekly step goals on log sheet and request a copy be sent to researchers weekly; Guide in monitoring step counts of each program walk with log sheet and request a copy be sent to researchers weekly. Guide in planning walks (specifying time, place and steps to walk) using a weekly planner.	Self-control
Walking guide; one-on-one consultations	Self-talk	Provide techniques for replacing negative self-statements with positive ones.	Emotional-coping responses

^a^The Walking Guide was a 27-page booklet developed for the Stepping Out program. The Walking Guide, a pedometer, log books, and weekly planners were distributed at the Week 6 session. One-on-one consultations occurred immediately following the assessments at Weeks 6, 12, and 24.

OA = osteoarthritis.

Participants received the program materials and instructions for following the program and wearing the pedometer after the assessment portion of the Week 6 session. The first author (NTMN, a doctoral student with training in exercise science and physical activity behaviour change) served as both data collector and interventionist. At that session, participants were asked to initially walk at least 1500 steps (approximately 15 minutes) on each 'walking' day in addition to any walking they were currently doing, and to do this additional walking in a single session. They were asked to increase from 1500 steps to 3000 steps (approximately 30 minutes) by the Week 12 assessment and, to accommodate participants who were unable to walk this amount continuously, were advised that the walks could be done in bouts of at least 1500 steps each. They were also advised to increase their step counts at a rate that was comfortable for them. At the Week 12 session, participants were asked to increase their walking to 6000 steps (approximately 60 minutes) by Week 18, the end of the intervention. At the Week 18 session, they were advised to either continue with the walking program or to try other physical activities of their choice for the last six weeks of the study, the follow-up period.

### Statistical analysis

Study completers were compared with those who dropped out of the study, using demographic and outcome variables measured at baseline. Likewise, the three-day and five-day walking groups were compared at baseline. Categorical variables were examined using the chi-squared test for independence, and continuous variables were examined with the Mann Whitney test, because the data were not normally distributed. For the Mann Whitney test, differences in the ranked positions of scores in different groups are compared [49].

Compliance with the study protocol's recommendation for GS intake, for the number of 'walking' days per week, and for the number of steps to walk each 'walking day' were computed using data collected from weekly log sheets. For each week between baseline and Week 18, GS compliance was defined as the proportion of participants who recorded taking two GS capsules per day at least five days of the week. For each week between Weeks 6 and 18, compliance with the number of walking days was defined as the proportion of participant who reported walking the prescribed number of days (three for the three-day walking group; five for the five-day walking group). Compliance with the number of steps prescribed for each walking day was defined as the proportion of participants who reported walking 1500 steps at Week 7 (after the first week of walking), 3000 steps at Week 12 and 6000 steps at Week 18. Chi-squared test for independence was used to compare groups on the proportion of participants who complied with the recommendation for GS intake each week. Independent samples *t*-tests were used to compare groups on the mean number of days walked during each of the 12 weeks of the Stepping Out program and on the mean number of steps walked per 'walking' day during that time. Type and usage of rescue analgesia were also collected from weekly log sheets, and median number of days that these medications were used over the intervention period was computed.

The Mann Whitney test was used to examine differences between the three-day and five-day walking groups at Weeks 6, 12, 18 and 24 for the main outcome variables, physical activity and OA symptoms. The remaining analyses were then analysed separately by group, only if group differences were found. Otherwise, data from the two groups were pooled for analysis of intervention effects. Differences between assessment weeks in scores on all outcome variables were examined using the Wilcoxon Signed Ranks Test. An effect size (r; z-score divided by the square root of the sample size) was computed for each statistically significant finding [49], and Cohen's d benchmark was used to determine the magnitude of the effect, with 0.20 representing small, 0.50 representing moderate and 0.80 representing large effect sizes [50]. Confidence intervals for the effect sizes were not calculated because data were not normally distributed. Instead, inter-quartile ranges of the raw scores were computed. Given that this was a feasibility study, data were analysed on a per protocol basis, meaning that participants who did not complete all study assessments were excluded. For study completers, missing data were replaced by the mean of the preceding and proceeding values [51]. Statistical significance was set at a two-tailed alpha level of 0.05 for all analyses.

## Results

### Participants

**Figure 2:**
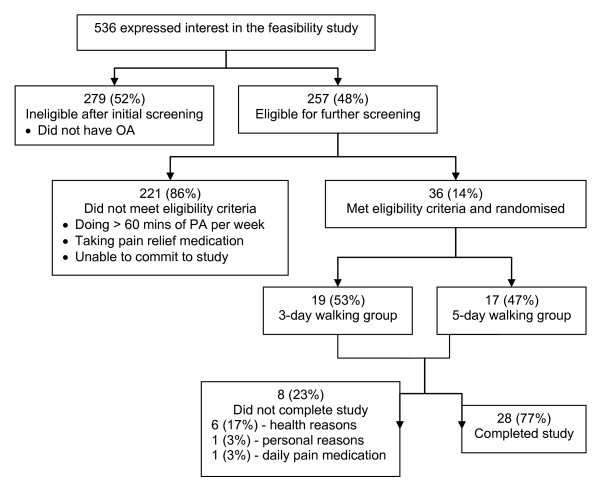
**Process of recruitment for the study**. OA, osteoarthritis. PA, physical activity.

Over 16 weeks of recruitment, 536 people expressed interest in the study (Figure 2). The preliminary screening revealed that 48% had physician-diagnosed OA in a knee or hip. Of these, 14% met all eligibility criteria, gave written informed consent and were enrolled into the study. Of those who met the eligibility criteria, 47% (n = 17) were randomised to the five-day walking group and 53% (n = 19) to the three-day walking group. Of the participants who enrolled, 77% completed the study (three-day group: n = 13, five-day group: n = 15). Three participants dropped out during the first six weeks of the study, before the walking program began. Reasons were a death in the family (n = 1), a physician's advice to withdraw due to potential impact of walking on OA (n = 1) and a physician's advice to withdraw due to potential impact of walking on other health conditions (n = 1). Five additional participants dropped out during the walking program. Reasons for drop-out from the three-day walking group were a death in the family (n = 1; dropout in Week 8), pain in the knees (n = 1; Week 7) and a torn Achilles tendon (n = 1; Week 7), and from the five-day walking groups were pain while walking due to leg length discrepancies (n = 1; Week 12) and development of Bakers' Cyst causing pain while walking (n = 1; Week 9). None of these conditions was directly attributable to participation in the program. No differences were found between study completers and those who dropped out on any study variable.

Demographic characteristics of study completers are presented in Table 2. Intervention groups did not differ significantly on any of the variables examined.

**Table 2:** Baseline demographic characteristics of participants who completed the study

	3-day walking group	5-day walking group	Total
	n = 13	n = 15	n = 28
	n (%)	n (%)	n (%)
**Sex**			
Men	6 (46)	5 (33)	11 (39)
Women	7 (54)	10 (67)	17 (61)
**Age (years)**			
40-59	4 (31)	7 (47)	11 (39)
60-75	9 (69)	8 (53)	17 (61)
**BMI (kg/m^2^)**			
<25	3 (23)	3 (20)	6 (21)
>25	10 (77)	12 (80)	22 (79)
**Marital status**			
Married or common-law relationship	9 (69)	9 (60)	18 (64)
Single	4 (31)	6 (40)	10 (36)
**Highest educational level achieved**			
High school degree or less	5 (39)	6 (40)	11 (39)
Schooling beyond high school	8 (61)	9 (60)	17 (61)
**Current employment status**			
Employed	7 (54)	6 (40)	13 (46)
Not employed	6 (46)	9 (60)	15 (54)
**Main lifetime occupation**			
Manager or professional	8 (61)	4 (27)	12 (43)
Other	5 (39)	11 (73)	16 (57)

Note: No significant differences were found between groups for any demographic variable (*P *> 0.05). BMI = body mass index.

### Compliance

From baseline to Week 18, 100% of three-day group participants were compliant with taking the weekly GS supplementation for all but three weeks, and 100% of five-day group participants were compliant with taking the weekly GS supplementation for all but two weeks. For weeks in which compliance was not 100%, compliance was 90% or more for each intervention group. No differences were found between groups in the proportion who were compliant with taking the GS (*P *= 0.18).

Nineteen of the 28 study participants (three-day group n = 7, 58%, five-day group n = 12, 80%) reported taking paracetamol and/or NSAIDs as rescue analgesia, with the most popular medications being paracetamol preparations (n = 12). Over the 18-week intervention period, study participants took rescue analgesia a median of 5.5 days (25^th ^percentile = 0 days; 75^th ^percentile = 18 days).

**Figure 3:**
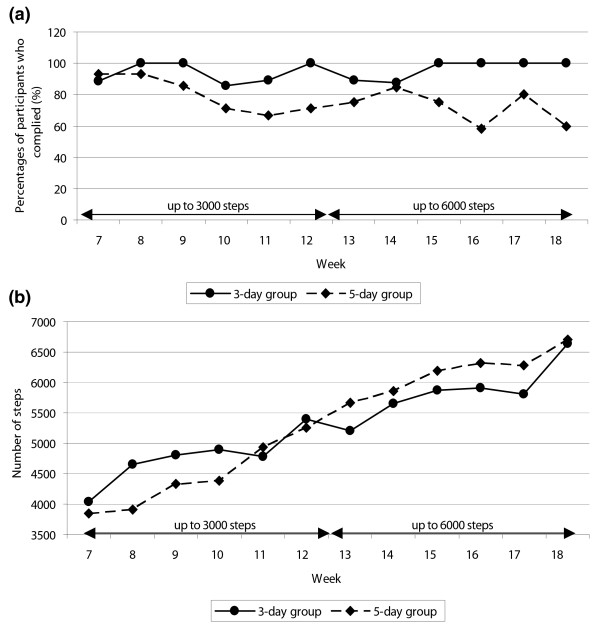
**Compliance with the Stepping Out program**. **(a) **The percentage of participants who complied with the number of 'walking' days per week of the walking program (Weeks 7 to 18 of the study). **(b) **Mean number of steps walked each 'walking' day during the 12-week Stepping Out program (Weeks 7 to 18 of the study).

For each week of the Stepping Out program (Weeks 7 to 18), most participants in both groups were compliant with walking the number of 'walking days' called for in the protocol (i.e., they walked the prescribed three or five days per week), but compliance was higher in the three-day walking group than in the five-day walking group (Figure 3). Among participants in the three-day walking group, there was 100% compliance with walking three days per week during Weeks 8, 9, 12, 15, and 18. Among participants in the five-day walking group, compliance ranged from 93% (Week 7) to 58% (Week 16) during the 12-week walking program. The mean number of days walked throughout the 12 weeks was also computed. No significant difference in number of days walked were found between groups although there was a trend in significance (*P *= 0.06). On average, participants in the three-day group walked three days per week (mean days/week = 3.07 (standard deviation (SD) 0.82) days), but participants in the five-day group did not walk five days per week (mean days/week = 3.93 (SD 1.09) days).

Another measure of compliance was the proportion of participants in each group who complied with the number of steps indicated in the study protocol. In the first week of the walking program (Week 7), 89% of participants in the three-day group and 93% in the five-day group complied with walking at least 1500 steps on each walking day. These percentages decreased to 75% in the three-day group and 79% in the five-day group by Week 12 when the target step level increased to 3000 steps. By Week 18, when the target step level increased to 6000 steps, the percentages were 83% and 50% in the three- and five-day groups, respectively. Participants in both groups increased the number of steps they walked each 'walking' day over the weeks of the Stepping Out program, and no significant group differences in steps per 'walking' day were seen. For the two groups combined, the mean number of steps walked per 'walking' day for the study increased from 3920 (SD 2441) per day during the first week of the walking program (Week 7) to 6683 (SD 3403) per day during the final week of the program (Week 18).

### Differences between groups

No significant differences were found between groups for the main outcome variables at any assessment week. Therefore, data from both groups were combined for the rest of the analyses. The only missing data were for weight and body mass index (BMI) for one person in Week 12, and for weight and BMI (n = 2), blood pressure (n = 2), post-SPS WOMAC pain (n = 3) and SPS (n = 3) at Week 18. Changes in outcome variables between Week 6 and Week 24 are shown in Tables 3 and 4. Changes from baseline to Week 6 (GS supplementation only) and from Week 6 to Week 24 (onset of walking program to end of follow-up) are described below. We chose to focus on Weeks 6 to 24 because, from a public health point of view, it is important to ascertain whether any effects are maintained after the end of the program.

**Table 3:** Median scores and interquartile ranges for the main study outcomes

		Week						
		
		0	6	12	18	24	6-24	
**Physical activity (min/week)**	**25^th^**	20.00	30.00	150.00	197.50	120.00	***z***	2.88
	**Median**	55.00	100.00^a^	225.00^b^	352.50^c^	190.00^d^	***P***	<0.001
	**75^th^**	108.75	221.25	360.00	555.00	405.00	***r***	0.38

**Physical function** **Self-paced step test (seconds)**	**25^th^**	92.50	75.00	68.25	60.50	58.50	***z***	-4.62
	**Median**	104.00	90.50^a^	79.50^b^	73.50^c^	70.00^d^	***P***	<0.001
	**75^th^**	129.75	102.50	90.25	81.50	75.75	***r***	0.62

**WOMAC measures**								
**Arthritis pain**	**25^th^**	11.00	8.00	5.00	5.25	3.25	***z***	-2.61
	**Median**	15.50	15.00	12.50^b^	12.00	9.00	***P***	0.01
	**75^th^**	24.75	24.75	19.00	19.00	14.00	***r***	0.35

**Stiffness**	**25^th^**	7.25	3.50	3.25	4.00	3.25	***z***	-1.87
	**Median**	10.00	7.00^a^	7.00	5.50	5.00	***P***	0.06
	**75^th^**	14.00	12.00	11.00	10.75	9.50	***r***	0.25

**Physical function**	**25^th^**	44.25	26.50	13.00	12.00	15.75	***z***	-3.11
	**Median**	63.50	58.00^a^	36.50^b^	33.00	35.00	***P***	<0.001
	**75^th^**	86.50	78.25	80.50	60.50	57.00	***r***	0.42

**Pain after step test**	**25^th^**	9.25	6.25	1.50	1.88	0.25	***z***	-3.11
	**Median**	15.00	11.50	7.50^b^	8.50	4.50	***P***	<0.001
	**75^th^**	24.75	22.00	16.00	15.00	13.25	***r***	0.42

**Total index**	**25^th^**	61.50	36.50	29.00	23.13	23.50	***z***	-2.95
	**Median**	89.50	77.50^a^	53.00^b^	48.00	51.50	***P***	<0.001
	**75^th^**	123.75	119.00	111.50	85.25	80.00	***r***	0.39

Note. Z scores, *P*-values and effect sizes (r) for changes between Weeks 6 and 24 are shown in the right hand column.

^a ^Week 6 significantly different from Week 0.

^b ^Week 12 significantly different from Week 6.

^c ^Week 18 significantly different from Week 12.

^d ^Week 24 significantly different from Week 18.

*r *= Effect size; WOMAC = Western Ontario and McMaster Universities Osteoarthritis Index.

**Table 4:** Median scores, interquartile range for secondary outcome variables

		Week						
		
		0	6	12	18	24	6-24	
**Arthritis self-efficacy measures**								
**Pain**	**25^th^**	45.00	53.50	58.50	60.50	70.00	***z***	2.64
	**Median**	55.00	69.00^a^	72.00	71.00	80.00^d^	***P***	0.01
	**75^th^**	72.00	75.50	86.00	90.00	91.50	***r***	0.35

**Function**	**25^th^**	58.89	68.61	72.50	74.17	73.61	***z***	1.91
	**Median**	74.44	79.44	86.67	88.33	88.89	***P***	0.06
	**75^th^**	89.44	89.72	94.72	92.22	94.17	***r***	0.26

**Other symptoms**	**25^th^**	43.75	57.08	70.42	73.33	78.75	***z***	-2.96
	**Median**	61.67	68.33^a^	78.33^b^	83.33	85.83^d^	***P***	<0.001
	**75^th^**	80.00	86.25	87.92	89.58	90.00	***r***	0.40

**Correlates of physical activity measures**								
**Self-regulation**	**25^th^**	23.25	19.50	34.00	33.38	30.75	***z***	3.69
	**Median**	30.00	27.00	38.00^b^	39.00	39.00	***P***	<0.001
	**75^th^**	34.00	36.50	45.00	48.25	47.25	***r***	0.49

**Self-efficacy**	**25^th^**	2.40	2.40	2.65	2.80	2.45	***z***	1.79
	**Median**	2.80	3.00	3.10^b^	3.20	3.30	***P***	0.07
	**75^th^**	3.20	3.35	3.55	3.60	3.75	***r***	0.24

**Benefits**	**25^th^**	53.00	53.25	54.25	53.00	53.00	***z***	-0.69
	**Median**	56.00	56.00	57.00	56.00	54.50	***P***	0.49
	**75^th^**	62.50	63.00	65.00	64.00	64.25	***r***	0.09

**Barriers**	**25^th^**	0.87	0.78	0.62	0.48	0.48	***z***	-2.57
	**Median**	1.48	1.04^a^	1.02	0.86	0.98	***P***	0.01
	**75^th^**	2.03	1.63	1.46	1.55	1.47	***r***	0.34

**Anxiety and depression (Goldberg)**	**25^th^**	4.25	1.25	2.00	2.00	1.00	***z***	-1.73
	**Median**	6.00	5.00	4.00	5.00	3.00	***P***	0.08
	**75^th^**	8.75	7.75	5.75	6.00	5.75	***r***	0.23

**Weight (kg)**	**25^th^**	69.25	69.88	72.00	69.38	69.00	***z***	-1.57
	**Median**	79.50	80.00	81.00^b^	79.25^c^	79.50^d^	***P***	0.11
	**75^th^**	95.50	97.00	94.00	89.13	91.75	***r***	0.21

Note. Z scores, *P*-values and effect sizes (r) for changes between Weeks 6 and 24 are shown in the right hand column.

^a ^Week 6 significantly different from Week 0.

^b ^Week 12 significantly different from Week 6.

^d ^Week 24 significantly different from Week 18.

*r *= Effect size.

### Changes between baseline and Week 6 (GS supplementation only)

Although instructed not to increase their physical activity, from baseline to the Week 6 assessment, participants significantly increased their median weekly minutes of physical activity (Table 3). There were also significant improvements (decreases) in SPS times and WOMAC stiffness and physical function scores although WOMAC pain scores did not change significantly (Table 3). Scores on the Arthritis Self-Efficacy Scale pain and 'other symptom' subscales and on the Barriers to Physical Activity Scale also improved significantly (Table 4).

### Changes between Week 6 and Week 24

Between the start of the walking program (Week 6) and the end of the follow-up period (Week 24), there were significant improvements in participants' weekly median minutes of physical activity, in SPS test times and in all WOMAC scores except stiffness scores (Table 3). However, there was a trend for improvement in stiffness (*P *= 0.06). Significant improvements were also seen in self-efficacy towards managing arthritis pain and 'other symptoms', in physical activity self-regulation, and in the number of perceived barriers to physical activity (Table 4). There were also trends for improvements in self-efficacy towards managing arthritis-related functioning (*P *= 0.06), in self-efficacy towards physical activity (*P *= 0.07) and in symptoms of anxiety and depression (*P *= 0.08).

## Discussion

The main aims of this feasibility study were to evaluate the combined effects of a progressive walking program and GS intake on symptoms of OA and on physical activity participation in people with hip and knee OA, and to compare the effectiveness of two frequencies (three and five days per week) and three steps levels (1500, 3000 and 6000 steps) of walking. Thirty-six participants were given GS for 18 weeks of the study. After the first six weeks, they began the 12-week graduated Stepping Out walking program and were randomised to walk three or five days per week.

For the first six weeks, before the introduction of Stepping Out, daily GS supplementation was found to be effective in alleviating symptoms of hip and knee OA. Stiffness and physical function, both measured with WOMAC subscales, improved significantly (median scores improved by 30% and 9%, respectively) although pain, also measured with the WOMAC, did not. Objectively-measured physical function also improved significantly, by 13%. It is possible that these changes were due to increases in physical activity in this period, even though participants were asked to not change their physical activity during this time. The improvements partially support those from previous randomised controlled trials. In these trials [23,24,52], improvements were significantly greater for the groups assigned to receive GS than for the groups assigned to receive placebos or alternative therapies. In a three-year trial, Reginster and colleagues [24] found that among patients with knee OA, WOMAC index scores improved 24% with daily GS supplementation and WOMAC physical function scores improved by 22%. Scores on the WOMAC pain scale also improved, by 19%. In a three-year trial by Pavelka and colleagues [23], patients with knee OA who took GS experienced improvements in pain and physical function of 20 to 25%. In a six-month trial [52], patients with knee OA who were assigned to a GS group had improvements in WOMAC index scores of 12% and physical function scores of 13%. In other trials [27,28,53], however, no significant improvements with glucosamine supplementation were found. Differences in findings between studies can be explained in part by the participant characteristics of each sample. In the studies that found no improvements, participants tended to have mild symptoms of OA at baseline. In the current study and in other studies that found significant improvements with GS, participants tended to have moderate to moderately-high levels of symptoms (i.e., median scores above the median point in the scale) at baseline. Other differences between studies include the GS preparation used. The bioavailability of GS products can affect the rate that the ingested GS reaches the target tissue to evoke metabolic changes in the articular cartilage [53]. This is the first time that the benefits of GS have been shown in a relatively short six-week period.

The major finding of the current study was that being encouraged to walk five days a week was not more effective than being encouraged to walk three days, in terms of increasing time spent in physical activities, reducing pain and stiffness, increasing physical function, and improving most other measures used in the study. These findings are not surprising given that at each follow up the three-day and five-day walking groups did not differ significantly in the mean number of days actually walked per week, the mean number of daily steps walked on each walking day as measured with a pedometer or their weekly minutes of physical activity as measured by questionnaire. On average, participants in the three-day walking group walked three days per week and participants in the five-day walking group walked slightly less than four days per week, suggesting that it may be difficult to get people with hip or knee OA to walk more than three to four days per week. Another important finding was that increasing the number of steps per walking day from 1500 to 3000 steps per day, in conjunction with GS intake, resulted in a 125% increase in minutes of physical activity, a 17% reduction in pain scores, and improvements in physical function measured subjectively using the WOMAC (a 37% reduction in scores) and objectively using the SPS test (a 12% decrease in time). When the walking step level was increased from 3000 to 6000 steps a day, the primary changes were a 57% increase in physical activity participation and an 8% improvement in objectively-measured physical function. During the six-week follow up, minutes per week of physical activity decreased 46%, but physical function, measured objectively, continued to improve significantly, by 5%. These results suggest that increasing walking by 3000 steps (about 30 minutes) on at least three days per week can significantly reduce pain and increase physical function in people with hip or knee OA and that increasing walking from 3000 steps to 6000 steps on each walking day may offer additional significant improvements in physical function only. In short, more benefit comes from increasing walking by 3000 than from increasing walking by another 3000 steps to 6000 steps.

The improvements in WOMAC pain and physical function are consistent with those reported for randomised controlled trials [14,15,44,54,55]. Kovar and colleagues [14] examined the effectiveness of an eight-week supervised walking and patient education program for 102 participants with knee OA. Participants who received the program exhibited a significant 27% decrease in arthritis pain and a non-significant 39% improvement in physical function. Similar results were reported by Evcik and Sonel [54] for a three-month randomised controlled trial with 90 participants with knee OA. Participants in a walking group had significant improvements ranging from 51% to 55% on the WOMAC pain subscale and of 57% on the WOMAC physical function subscale, compared with controls. In the most recent study, 34 older adults with knee OA were randomised to receive a pedometer-based walking program with arthritis self-management education or the education only [55]. Participants who received the walking program had an improvement of 10% in pain while the education-only participants reported no improvement in pain. Differences between groups were not statistically significant, possibly due to a lack of power to detect differences because of the small sample size.

Differences in effects among studies are likely to be due to differences in a number of factors, including sample size; eligibility criteria, most notably the criteria for exclusion based on baseline physical activity levels and on measures used to determine OA status; length of the intervention period; the physical activity prescription; the attention from and contact with intervention staff; the mode of intervention delivery; the type of 'rescue medications' allowed during the trial; and the choice of outcome measures. Compared with other studies, the current study was short (18 weeks) and included a small sample. However, it was a feasibility designed to inform a larger study. In previous studies, protocols ranged from supervised exercise in a hospital [14] to unsupervised exercise with weekly phone calls [54]. Some included both supervised and unsupervised exercise [15]. Some studies prescribed a mix of aerobic and strength training exercises [15,56,57], and others prescribed just aerobic activity [22,55]. In the current study, the exercise program was unsupervised walking and contact time with the participants was minimal, in order to test the effectiveness of a program that could be widely disseminated. Another difference was that in the current study, strict eligibility criteria were used to guarantee that only OA sufferers most in need of a physical activity program (i.e., those who were engaging in no or low levels of physical activity) were included. Other studies did not have exclusion criteria based on physical activity [54,55]. Participants in the current study were allowed rescue analgesia as needed while some previous studies limited these to a maximum dose of 4000 mg per day of paracetamol [27,53]. The highest dosage of rescue analgesia recorded by participants in our study was 2000 mg per day of paracetamol, which was taken by 63% (n = 12) of those who took rescue medications.

### Strengths

This study was the first to look at the effectiveness of different frequencies and step levels of walking in combination with GS for relieving symptoms of OA. Although the study included only a small sample, the findings provide preliminary evidence on the number of walking steps needed to relieve OA symptoms and on the effects of different step levels on OA symptoms. Importantly, physical function was assessed with the SPS test, an objective measure, to verify any improvements found in physical function scores on the WOMAC, and walking behaviour was measured objectively with pedometers. Other instruments used in the study were also validated measures commonly used in the physical activity field. Another strength was that behaviour change theory and the empirical literature were used to develop the intervention strategies and content. Previous studies have indicated that programs designed to impact self-efficacy can have beneficial effects for individuals with OA [57-59], and thus strategies were developed to positively impact self-efficacy in this study. For example, a pedometer was provided as a self-monitoring and motivational tool, and monitoring step counts made it possible to objectively assess the amount of walking achieved by each participant. Recording the number of steps on weekly log sheets provided important information on participants' progress and compliance with the walking program. Only one previous study [55] has used pedometers to increase physical activity among individuals with OA. Hence, this feasibility study can help inform future research examining the use of pedometers for increasing physical activity among individuals with knee or hip OA. Most studies have looked at older (65+ years) adults with OA, but this feasibility study included younger participants aged 40+ years, making the findings and the self-directed intervention relevant to working adults. A final strength was that the program was home-based and unsupervised, to accommodate the schedules, symptoms and walking ability of different participants. This made the program easier to integrate into individuals' lifestyles than group-based activities. Anecdotally, several participants indicated that they would not have been able to follow the program if it had involved regular attendance at a class.

### Limitations

The main limitation of the current study was the small sample size. Difficulties with recruitment, as well as loss to follow up, resulted in a small analysis sample. Some of the loss to follow up was attributable to pre-existing conditions that aggravated symptoms during the walking program. However, two participants who dropped out reported that their physicians advised against walking. Also of note is that it is likely that a number of analyses lacked power to detect differences between the two walking groups. In addition, participants in the two groups reported walking approximately the same number of days per week and spending similar minutes per week in physical activities, making group differences in the other outcome measures unlikely. Another limitation was the use of self-report data. Although some studies use x-rays to assess changes in OA progression, this study was too short to expect to see changes with x-rays. Therefore, the primary outcome measure was the WOMAC, which has been validated and is widely used to examine the effects of exercise on OA symptoms [34,35]. It should also be noted that most previous studies measured joint space narrowing to assess the effectiveness of GS and exercise for OA sufferers [23,24,28]. This was not measured in the current study due to the short intervention period of 18 weeks. We cannot, therefore, conclude that a combination of walking and GS supplementation will slow down joint space narrowing, even though there was relief of OA symptoms.

Given that previous research has already shown that exercise is beneficial for individuals with OA [60-63], the main aim of the current study was to compare the effectiveness of two walking programs in combination with GS, rather than to compare walking to no walking. Therefore, although a larger sample size would have allowed for the inclusion of a no-walking control group, the inclusion of such a control group was not essential to meet the main aim. Without a placebo control, however, the study was unable to determine the effects of GS compared with placebo during the first six weeks of the intervention and the effects of the walking program without the use of GS during the remaining weeks. Furthermore, the assessor and main analyst (NTMN) was not blinded to group allocation and conducted the randomisation process before baseline, which may have contributed to ascertainment or performance biases. The current study used a per-protocol analysis but an intent-to-treat analysis should be used for a future study with a larger sample size. Another minor limitation was that the weather may have been a potential confounder. However, participants continued to walk consistently throughout the 18-week walking program and six-week follow up. Finally, it was not possible to obtain radiographic evidence from all the participants to confirm diagnosis and severity of their OA. Participants who were unable to provide radiographic evidence obtained a letter of diagnosis from their physician, but severity of the disease was not confirmed. However, from WOMAC scores obtained at baseline, participants were found to have, on average, moderate symptoms of knee or hip OA.

## Conclusions

Although the study included a small sample, the findings provide preliminary evidence that OA sufferers can obtain health-related benefits from the combination of GS and walking. Walking 3000 steps per day for exercise, in bouts of at least 1500 steps each, on at least three days per week, provided these benefits. This amount of walking is less than current physical activity recommendations for the general population, but follows the recommendations for people with arthritis [19]. The study also provides support for the acceptability of GS in conjunction with a home-based walking program for people with OA, as participants were willing to comply with taking the glucosamine twice daily, wearing a pedometer, completing log sheets, walking three days per week and progressively increasing their steps per 'walking' day. With positive results, there is a need now to conduct a larger placebo-controlled trial to strengthen the findings and establish definitive data on the efficacy of the Stepping Out program. If the benefits of this program are confirmed, it could be promoted to increase physical activity among people with hip or knee OA.

## Abbreviations

BMI: body mass index; ES: effect size; GS: glucosamine sulphate; NSAIDs: nonsteroidal anti-inflammatory drugs; OA: osteoarthritis; SD: standard deviation; SPS: self-paced step test; WOMAC Osteoarthritis Index: Western Ontario and McMaster Universities Osteoarthritis Index.

## Competing interests

All authors declare that they have no competing interests. Sanofi-Aventis Consumer Health Care supplied the GS for use in this study. The organisation therefore would like to see the results of the study published but they had no input into the study design or implementation.

## Authors' contributions

NTMN, KCH and WJB participated in the study conception and design. NTMN participated in the recruitment of participants, data acquisition, implementation of the intervention and statistical analysis. KCH participated in the recruitment of participants, the oversight of study implementation and the statistical analysis. WJB participated in the oversight of study implementation and the statistical analysis. All authors participated in the interpretation of the data and the drafting of the manuscript, and all authors read and approved the final manuscript.
